# *N*′-(3,4-Dimeth­oxy­benzyl­idene)benzo­hydrazide

**DOI:** 10.1107/S1600536810052128

**Published:** 2010-12-18

**Authors:** Saeedeh Hashemian, Vahideh Ghaeinee, Behrouz Notash

**Affiliations:** a Department of Chemistry, Islamic Azad University, Yazd Branch, Yazd, Iran; b Department of Chemistry, Shahid Beheshti University, G.C., Evin, Tehran 1983963113, Iran

## Abstract

The crystal structure of the title Schiff base compound, C_16_H_16_N_2_O_3_, is characterized by chains of mol­ecules linked by inter­molecular N—H⋯O hydrogen bonds running along the *c* axis. Further stabilization is provided by weak C—H⋯O contacts. The dihedral angle between the aromatic rings is 38.31 (7)°.

## Related literature

For related structures see: Alhadi *et al.* (2009 ▶); Das & Pal (2004 ▶); Tamboura *et al.* (2009 ▶); Zhou *et al.* (2009 ▶).
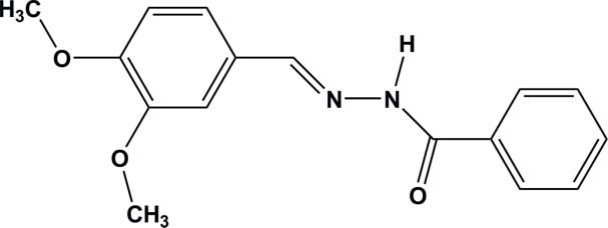


## Experimental

### 

#### Crystal data

C_16_H_16_N_2_O_3_
*M* *_r_* = 284.31
Monoclinic, 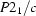

*a* = 12.612 (3) Å
*b* = 11.291 (2) Å
*c* = 9.892 (2) Å
β = 95.46 (3)°
*V* = 1402.3 (5) Å^3^
*Z* = 4
Mo *K*α radiation
μ = 0.09 mm^−1^
*T* = 120 K
0.5 × 0.4 × 0.15 mm

#### Data collection

Stoe IPDS II diffractometer
Absorption correction: numerical shape of crystal determined optically *T*_min_ = 0.954, *T*_max_ = 0.984
9840 measured reflections
3758 independent reflections
3054 reflections with *I* > 2σ(*I*)
*R*_int_ = 0.052

#### Refinement

*R*[*F* ^2^ > 2σ(*F* ^2^)] = 0.050
*wR*(*F* ^2^) = 0.131
*S* = 1.03
3758 reflections
200 parameters
H atoms treated by a mixture of independent and constrained refinement
Δρ_max_ = 0.36 e Å^−3^
Δρ_min_ = −0.24 e Å^−3^

### 

Data collection: *X-AREA* (Stoe & Cie, 2005 ▶); cell refinement: *X-AREA*; data reduction: *X-AREA*; program(s) used to solve structure: *SHELXS97* (Sheldrick, 2008 ▶); program(s) used to refine structure: *SHELXL97* (Sheldrick, 2008 ▶); molecular graphics: *ORTEP-3 for Windows* (Farrugia, 1997 ▶); software used to prepare material for publication: *WinGX* (Farrugia, 1999 ▶).

## Supplementary Material

Crystal structure: contains datablocks I, global. DOI: 10.1107/S1600536810052128/bt5434sup1.cif

Structure factors: contains datablocks I. DOI: 10.1107/S1600536810052128/bt5434Isup2.hkl

Additional supplementary materials:  crystallographic information; 3D view; checkCIF report

## Figures and Tables

**Table 1:** Hydrogen-bond geometry (Å, °)

*D*—H⋯*A*	*D*—H	H⋯*A*	*D*⋯*A*	*D*—H⋯*A*
C9—H9⋯O3^i^	0.957 (19)	2.455 (19)	3.2702 (18)	142.9 (15)
C7—H7*B*⋯O3^ii^	0.96	2.40	3.2434 (18)	146
N2—H2*A*⋯O3^i^	0.87 (2)	2.07 (2)	2.9219 (16)	163.2 (18)

Symmetry codes: (i) 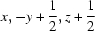; (ii) 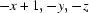.

**Fig. 1.**
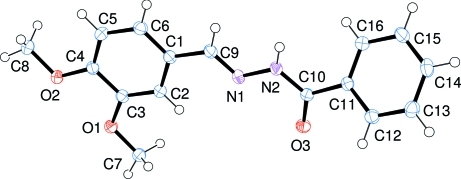
The molecular structure of N'-(3,4-dimethoxybenzylidene)benzohydrazide with displacement ellipsoids drawn at 50% probability level.

**Fig. 2.**
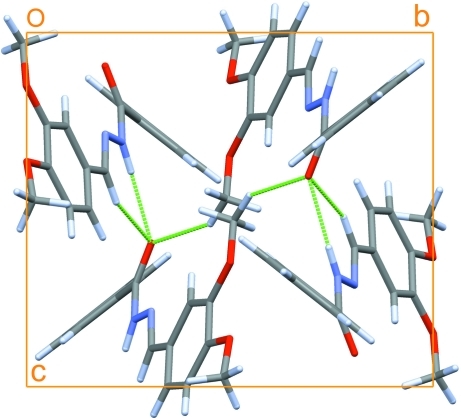
Packing diagram of the title compound viewed down the a axis. The intermolecular N—H···O and C—H···O hydrogen bonds are shown as green dashed lines.

## supplementary crystallographic information

### Comment

Schiff bases are important organic compounds. Metal complexes based on Schiff bases have received considerable attention because they can be utilized as a model of active centers in various complexes (Tamboura *et al.*, 2009; Das & Pal, 2004).

We report here the crystal structure of *N*'-(3,4-dimethoxybenzylidene)-benzohydrazide. The title compound was synthesized from the reaction of 3,4-dimethoxy benzaldehyde and benzoylhydrazine in methanol. The asymmetric unit of the title compound is shown in Fig. 1. The bond lengths and angles are comparable to those observed for *N*'-(2,4-dimethoxybenzylidene)-3,4,5-trihydroxybenzohydrazide (Alhadi *et al.*, 2009) and *N*'-(3,4-dimethoxybenzylidene)acetohydrazide (Zhou *et al.*, 2009). The crystal structure is characterized by chains of running along the c-axis. The molecules in a chain are linked by intermolecular N—H···O hydrogen bonds. Further stabilization is provided by weak C—H···O contacts.

### Experimental

To a methanol solution (20 ml) of benzoylhydrazine (1.0 mmol, 0.135 g), 3,4-dimethoxybenzaldehyde (1.0 mmol, 0.165 g), a few drops of acetic acid were added. The mixture was refluxed for 6 h and then cooled to room temperature. The white crystalline solid was collected by filtration, washed with cold methanol and finally dried in air (m.p: 181–183 °C, yield: 81%). Single crystals suitable for X-ray analysis were obtained by slow evaporation of an ethanol solution at room temperature.

### Refinement

The hydrogen atom bonded to N and to the imine C atom were found in difference Fourier map and refined isotropically without restraint. The C—H protons were positioned geometrically and refined as riding atoms with C—H = 0.93 Å and *U*iso(H) = 1.2 *U*eq(C) for aromatic C—H   and C—H = 0.96 Å and *U*iso(H) = 1.5 *Ueq*(C) for methyl groups.

### Crystal data

**Table:**

C_16_H_16_N_2_O_3_	*F*(000) = 600
*M**_r_* = 284.31	*D*_x_ = 1.347 Mg m^−^^3^
Monoclinic, *P*2_1_/*c*	Mo *K*α radiation, λ = 0.71073 Å
Hall symbol: -P 2ybc	Cell parameters from 3758 reflections
*a* = 12.612 (3) Å	θ = 2.4–29.2°
*b* = 11.291 (2) Å	µ = 0.09 mm^−^^1^
*c* = 9.892 (2) Å	*T* = 120 K
β = 95.46 (3)°	Plate, colorless
*V* = 1402.3 (5) Å^3^	0.5 × 0.4 × 0.15 mm
*Z* = 4	

### Data collection

**Table:**

Stoe IPDS II diffractometer	3758 independent reflections
Radiation source: fine-focus sealed tube	3054 reflections with *I* > 2σ(*I*)
graphite	*R*_int_ = 0.052
Detector resolution: 0.15 mm pixels mm^-1^	θ_max_ = 29.2°, θ_min_ = 2.4°
rotation method scans	*h* = −17→17
Absorption correction: numerical shape of crystal determined optically	*k* = −13→15
*T*_min_ = 0.954, *T*_max_ = 0.984	*l* = −13→13
9840 measured reflections	

### Refinement

**Table:**

Refinement on *F*^2^	Primary atom site location: structure-invariant direct methods
Least-squares matrix: full	Secondary atom site location: difference Fourier map
*R*[*F*^2^ > 2σ(*F*^2^)] = 0.050	Hydrogen site location: inferred from neighbouring sites
*wR*(*F*^2^) = 0.131	H atoms treated by a mixture of independent and constrained refinement
*S* = 1.03	*w* = 1/[σ^2^(*F*_o_^2^) + (0.068*P*)^2^ + 0.4464*P*] where *P* = (*F*_o_^2^ + 2*F*_c_^2^)/3
3758 reflections	(Δ/σ)_max_ = 0.001
200 parameters	Δρ_max_ = 0.36 e Å^−^^3^
0 restraints	Δρ_min_ = −0.24 e Å^−^^3^

### Special details

**Table:**

Geometry. All e.s.d.'s (except the e.s.d. in the dihedral angle between two l.s. planes) are estimated using the full covariance matrix. The cell e.s.d.'s are taken into account individually in the estimation of e.s.d.'s in distances, angles and torsion angles; correlations between e.s.d.'s in cell parameters are only used when they are defined by crystal symmetry. An approximate (isotropic) treatment of cell e.s.d.'s is used for estimating e.s.d.'s involving l.s. planes.
Refinement. Refinement of *F*^2^ against ALL reflections. The weighted *R*-factor *wR* and goodness of fit *S* are based on *F*^2^, conventional *R*-factors *R* are based on *F*, with *F* set to zero for negative *F*^2^. The threshold expression of *F*^2^ > σ(*F*^2^) is used only for calculating *R*-factors(gt) *etc*. and is not relevant to the choice of reflections for refinement. *R*-factors based on *F*^2^ are statistically about twice as large as those based on *F*, and *R*- factors based on ALL data will be even larger.

### Fractional atomic coordinates and isotropic or equivalent isotropic displacement parameters (Å^2^)

**Table:**

	*x*	*y*	*z*	*U*_iso_*/*U*_eq_	
N2	0.77928 (9)	0.23464 (11)	0.31038 (12)	0.0204 (2)	
O2	0.21028 (8)	0.00051 (9)	0.36444 (11)	0.0261 (2)	
C10	0.84378 (10)	0.23202 (11)	0.20934 (13)	0.0184 (2)	
N1	0.67552 (9)	0.19349 (11)	0.28696 (12)	0.0213 (2)	
O1	0.30865 (8)	−0.00086 (9)	0.14959 (10)	0.0241 (2)	
C2	0.46487 (10)	0.09658 (12)	0.26416 (13)	0.0205 (3)	
H2	0.5013	0.0941	0.1867	0.025*	
C11	0.95522 (10)	0.27473 (12)	0.24654 (13)	0.0185 (3)	
C9	0.62290 (10)	0.19298 (12)	0.39172 (14)	0.0213 (3)	
C1	0.51348 (10)	0.14808 (12)	0.38429 (14)	0.0205 (3)	
C4	0.30798 (10)	0.05297 (12)	0.37871 (14)	0.0216 (3)	
C3	0.36354 (10)	0.04990 (12)	0.26093 (14)	0.0208 (3)	
C6	0.45799 (11)	0.15271 (13)	0.49842 (14)	0.0232 (3)	
H6	0.4895	0.1871	0.5777	0.028*	
C12	1.03648 (11)	0.21544 (13)	0.18854 (14)	0.0229 (3)	
H12	1.0200	0.1524	0.1298	0.028*	
C5	0.35478 (11)	0.10607 (13)	0.49557 (14)	0.0233 (3)	
H5	0.3177	0.1108	0.5723	0.028*	
C16	0.97968 (11)	0.37076 (13)	0.33195 (14)	0.0242 (3)	
H16	0.9258	0.4113	0.3702	0.029*	
C15	1.08551 (12)	0.40592 (14)	0.35984 (15)	0.0286 (3)	
H15	1.1021	0.4706	0.4162	0.034*	
C13	1.14179 (11)	0.24953 (13)	0.21765 (15)	0.0264 (3)	
H13	1.1958	0.2089	0.1797	0.032*	
C7	0.36427 (11)	−0.00915 (13)	0.03132 (14)	0.0246 (3)	
H7A	0.3818	0.0689	0.0021	0.037*	
H7B	0.3200	−0.0479	−0.0396	0.037*	
H7C	0.4285	−0.0540	0.0518	0.037*	
C14	1.16611 (11)	0.34472 (14)	0.30382 (15)	0.0278 (3)	
H14	1.2367	0.3676	0.3241	0.033*	
C8	0.14420 (12)	0.01622 (15)	0.47217 (16)	0.0305 (3)	
H8A	0.1791	−0.0161	0.5545	0.046*	
H8B	0.0776	−0.0238	0.4502	0.046*	
H8C	0.1313	0.0992	0.4844	0.046*	
O3	0.81626 (8)	0.19329 (9)	0.09466 (10)	0.0229 (2)	
H9	0.6538 (15)	0.2201 (17)	0.4783 (19)	0.030 (5)*	
H2A	0.8042 (16)	0.2565 (18)	0.392 (2)	0.036 (5)*	

### Atomic displacement parameters (Å^2^)

**Table:**

	*U*^11^	*U*^22^	*U*^33^	*U*^12^	*U*^13^	*U*^23^
N2	0.0185 (5)	0.0237 (6)	0.0189 (5)	−0.0038 (4)	0.0007 (4)	−0.0015 (4)
O2	0.0212 (5)	0.0279 (5)	0.0304 (5)	−0.0057 (4)	0.0089 (4)	−0.0066 (4)
C10	0.0200 (5)	0.0165 (6)	0.0187 (6)	0.0008 (4)	0.0010 (4)	0.0011 (5)
N1	0.0183 (5)	0.0223 (6)	0.0231 (5)	−0.0025 (4)	0.0001 (4)	−0.0003 (4)
O1	0.0200 (4)	0.0283 (5)	0.0240 (5)	−0.0045 (4)	0.0025 (4)	−0.0055 (4)
C2	0.0198 (6)	0.0214 (6)	0.0207 (6)	−0.0003 (5)	0.0032 (5)	−0.0005 (5)
C11	0.0194 (6)	0.0182 (6)	0.0177 (6)	−0.0011 (4)	0.0009 (4)	0.0031 (5)
C9	0.0202 (6)	0.0205 (6)	0.0230 (6)	−0.0016 (5)	0.0004 (5)	−0.0016 (5)
C1	0.0196 (6)	0.0188 (6)	0.0230 (6)	−0.0005 (5)	0.0014 (5)	−0.0004 (5)
C4	0.0194 (6)	0.0190 (6)	0.0267 (6)	−0.0009 (5)	0.0042 (5)	−0.0007 (5)
C3	0.0207 (6)	0.0193 (6)	0.0224 (6)	0.0000 (5)	0.0013 (5)	−0.0007 (5)
C6	0.0236 (6)	0.0233 (7)	0.0226 (6)	−0.0007 (5)	0.0013 (5)	−0.0021 (5)
C12	0.0229 (6)	0.0211 (6)	0.0248 (6)	0.0010 (5)	0.0023 (5)	−0.0004 (5)
C5	0.0231 (6)	0.0247 (7)	0.0230 (6)	0.0001 (5)	0.0067 (5)	−0.0013 (5)
C16	0.0240 (6)	0.0237 (7)	0.0252 (6)	−0.0021 (5)	0.0047 (5)	−0.0036 (5)
C15	0.0294 (7)	0.0306 (7)	0.0257 (7)	−0.0101 (6)	0.0025 (6)	−0.0053 (6)
C13	0.0215 (6)	0.0267 (7)	0.0315 (7)	0.0029 (5)	0.0041 (5)	0.0020 (6)
C7	0.0227 (6)	0.0274 (7)	0.0239 (6)	−0.0035 (5)	0.0029 (5)	−0.0044 (5)
C14	0.0196 (6)	0.0359 (8)	0.0274 (7)	−0.0068 (5)	−0.0004 (5)	0.0032 (6)
C8	0.0253 (7)	0.0340 (8)	0.0340 (8)	−0.0067 (6)	0.0127 (6)	−0.0073 (6)
O3	0.0236 (5)	0.0257 (5)	0.0188 (4)	−0.0011 (4)	−0.0005 (4)	−0.0013 (4)

### Geometric parameters (Å, °)

**Table:**

N2—C10	1.3477 (18)	C4—C3	1.416 (2)
N2—N1	1.3872 (15)	C6—C5	1.4020 (19)
N2—H2A	0.87 (2)	C6—H6	0.9300
O2—C4	1.3624 (16)	C12—C13	1.3865 (19)
O2—C8	1.4250 (18)	C12—H12	0.9300
C10—O3	1.2344 (16)	C5—H5	0.9300
C10—C11	1.4983 (17)	C16—C15	1.3947 (19)
N1—C9	1.2826 (19)	C16—H16	0.9300
O1—C3	1.3695 (16)	C15—C14	1.387 (2)
O1—C7	1.4233 (17)	C15—H15	0.9300
C2—C3	1.3800 (18)	C13—C14	1.388 (2)
C2—C1	1.4098 (18)	C13—H13	0.9300
C2—H2	0.9300	C7—H7A	0.9600
C11—C16	1.3910 (19)	C7—H7B	0.9600
C11—C12	1.3927 (19)	C7—H7C	0.9600
C9—C1	1.4655 (18)	C14—H14	0.9300
C9—H9	0.957 (19)	C8—H8A	0.9600
C1—C6	1.385 (2)	C8—H8B	0.9600
C4—C5	1.3836 (19)	C8—H8C	0.9600
			
C10—N2—N1	119.69 (11)	C13—C12—C11	120.60 (13)
C10—N2—H2A	120.2 (13)	C13—C12—H12	119.7
N1—N2—H2A	119.9 (13)	C11—C12—H12	119.7
C4—O2—C8	117.13 (11)	C4—C5—C6	120.07 (13)
O3—C10—N2	123.54 (12)	C4—C5—H5	120.0
O3—C10—C11	120.99 (12)	C6—C5—H5	120.0
N2—C10—C11	115.41 (11)	C11—C16—C15	119.66 (13)
C9—N1—N2	114.77 (11)	C11—C16—H16	120.2
C3—O1—C7	115.95 (10)	C15—C16—H16	120.2
C3—C2—C1	120.30 (13)	C14—C15—C16	120.20 (14)
C3—C2—H2	119.9	C14—C15—H15	119.9
C1—C2—H2	119.9	C16—C15—H15	119.9
C16—C11—C12	119.70 (12)	C12—C13—C14	119.60 (14)
C16—C11—C10	123.28 (12)	C12—C13—H13	120.2
C12—C11—C10	117.00 (12)	C14—C13—H13	120.2
N1—C9—C1	121.22 (12)	O1—C7—H7A	109.5
N1—C9—H9	121.5 (11)	O1—C7—H7B	109.5
C1—C9—H9	117.2 (11)	H7A—C7—H7B	109.5
C6—C1—C2	119.33 (12)	O1—C7—H7C	109.5
C6—C1—C9	119.52 (12)	H7A—C7—H7C	109.5
C2—C1—C9	121.11 (12)	H7B—C7—H7C	109.5
O2—C4—C5	125.78 (13)	C15—C14—C13	120.23 (13)
O2—C4—C3	114.69 (12)	C15—C14—H14	119.9
C5—C4—C3	119.53 (12)	C13—C14—H14	119.9
O1—C3—C2	125.07 (13)	O2—C8—H8A	109.5
O1—C3—C4	114.84 (12)	O2—C8—H8B	109.5
C2—C3—C4	120.09 (12)	H8A—C8—H8B	109.5
C1—C6—C5	120.65 (13)	O2—C8—H8C	109.5
C1—C6—H6	119.7	H8A—C8—H8C	109.5
C5—C6—H6	119.7	H8B—C8—H8C	109.5
			
N1—N2—C10—O3	−0.8 (2)	O2—C4—C3—O1	2.59 (17)
N1—N2—C10—C11	−178.05 (11)	C5—C4—C3—O1	−177.78 (12)
C10—N2—N1—C9	175.87 (12)	O2—C4—C3—C2	−177.78 (13)
O3—C10—C11—C16	143.82 (14)	C5—C4—C3—C2	1.9 (2)
N2—C10—C11—C16	−38.82 (18)	C2—C1—C6—C5	0.5 (2)
O3—C10—C11—C12	−34.55 (18)	C9—C1—C6—C5	−177.44 (13)
N2—C10—C11—C12	142.80 (13)	C16—C11—C12—C13	1.5 (2)
N2—N1—C9—C1	−177.84 (12)	C10—C11—C12—C13	179.95 (12)
C3—C2—C1—C6	−0.8 (2)	O2—C4—C5—C6	177.36 (13)
C3—C2—C1—C9	177.03 (12)	C3—C4—C5—C6	−2.2 (2)
N1—C9—C1—C6	−177.81 (13)	C1—C6—C5—C4	1.1 (2)
N1—C9—C1—C2	4.3 (2)	C12—C11—C16—C15	−0.7 (2)
C8—O2—C4—C5	9.6 (2)	C10—C11—C16—C15	−179.04 (13)
C8—O2—C4—C3	−170.84 (13)	C11—C16—C15—C14	−0.6 (2)
C7—O1—C3—C2	2.7 (2)	C11—C12—C13—C14	−1.0 (2)
C7—O1—C3—C4	−177.68 (12)	C16—C15—C14—C13	1.2 (2)
C1—C2—C3—O1	179.27 (13)	C12—C13—C14—C15	−0.4 (2)
C1—C2—C3—C4	−0.3 (2)		

### Hydrogen-bond geometry (Å, °)

**Table:**

*D*—H···*A*	*D*—H	H···*A*	*D*···*A*	*D*—H···*A*
C9—H9···O3^i^	0.957 (19)	2.455 (19)	3.2702 (18)	142.9 (15)
C7—H7B···O3^ii^	0.96	2.40	3.2434 (18)	146.
N2—H2A···O3^i^	0.87 (2)	2.07 (2)	2.9219 (16)	163.2 (18)

Symmetry codes: (i) *x*, −*y*+1/2, *z*+1/2; (ii) −*x*+1, −*y*, −*z*.
